# Matrix stiffness regulates nucleus pulposus cell glycolysis by MRTF-A-dependent mechanotransduction

**DOI:** 10.1038/s41413-025-00402-7

**Published:** 2025-02-14

**Authors:** Haoran Xu, Kang Wei, Jinhao Ni, Xiaofeng Deng, Yuexing Wang, Taiyang Xiang, Fanglong Song, Qianliang Wang, Yanping Niu, Fengxian Jiang, Jun Wang, Lei Sheng, Jun Dai

**Affiliations:** 1 Department of Orthopedic Surgery, The Second Affiliated Hospital of Soochow University, Suzhou, China; 2 Department of Orthopedic Surgery, Tongji Hospital, Tongji Medical College, Huazhong University of Science and Technology, Wuhan, China; 3 Department of Plastic Surgery, Zhongnan Hospital of Wuhan University, Wuhan, China; 4 Department of Rehabilitation, Tongji Hospital, Tongji Medical College, Huazhong University of Science and Technology, Wuhan, China; 5 State Key Laboratory of Oral Diseases, National Clinical Research Center for Oral Diseases, Department of Periodontics, West China Hospital of Stomatology, Sichuan University, Chengdu, China

**Keywords:** Metabolic disorders, Metabolism, Pathogenesis

## Abstract

Increased matrix stiffness of nucleus pulposus (NP) tissue is a main feature of intervertebral disc degeneration (IVDD) and affects various functions of nucleus pulposus cells (NPCs). Glycolysis is the main energy source for NPC survival, but the effects and underlying mechanisms of increased extracellular matrix (ECM) stiffness on NPC glycolysis remain unknown. In this study, hydrogels with different stiffness were established to mimic the mechanical environment of NPCs. Notably, increased matrix stiffness in degenerated NP tissues from IVDD patients was accompanied with impaired glycolysis, and NPCs cultured on rigid substrates exhibited a reduction in glycolysis. Meanwhile, RNA sequencing analysis showed altered cytoskeleton-related gene expression in NPCs on rigid substrates. Myocardin-related transcription factor A (MRTF-A) is a transcriptional coactivator in mechanotransduction mainly responding to cytoskeleton remodeling, which was activated and translocated to the nucleus under rigid substrate and was upregulated during IVDD progression. Furthermore, gas chromatography-mass spectrometry (GC-MS) analysis revealed that MRTF-A overexpression reduced NPC glycolytic metabolite abundance and identified a correlation with AMPK pathway. Mechanistically, rigid substrates and MRTF-A overexpression inhibited Kidins220 expression and AMPK phosphorylation in NPCs, whereas MRTF-A inhibition, treated with the MRTF-A inhibitor CCG, partially rescued NP tissue degeneration and glycolytic enzyme expression. Our data demonstrate that MRTF-A is a critical regulator that responds to increased matrix stiffness in IVDD, and MRTF-A activation reduces NPC glycolysis by down-regulating Kidins220 and inhibiting AMPK phosphorylation.

## Introduction

Intervertebral disc degeneration (IVDD) is increasingly prevalent in the population and is involved in various factors, including aging, overload, and obesity.^1,2^ The progression of IVDD is accompanied with a variety of symptoms such as low back pain, intervertebral disc herniation, spinal stenosis, and limb paralysis, resulting in poor life quality and large financial burden for patients.^3–5^ Traditional pharmacological and surgical therapies primarily focus on relieving symptoms of low back pain rather than the underlying etiology, and current therapies have a risk of recurrence and progression.^5,6^

The intervertebral disc (IVD) is comprised of nucleus pulposus (NP), annulus fibrosus, and cartilage endplate.^7^ Degeneration of NP tissue has been identified as the predominant pathological change in IVDD process. NP tissue is physiologically located in a hypoxic, acidic, and closed environment, and essential nutrients predominantly diffuse from the peripheral vasculature.^8,9^ Due to this special microenvironment, anaerobic glycolysis serves as the primary adenosine triphosphate (ATP) source for nucleus pulposus cells (NPCs).^10,11^ NPC degeneration is accompanied with insufficient glucose supply, lactic acid accumulation, and reduced glycolysis, thereby acting as a feedback loop to aggravate NPC degeneration and metabolic dysfunction.^11,12^ Nevertheless, the regulatory mechanism for abnormal glycolysis in degenerated NPCs remains to be further studied.

Numerous studies uncovered that degenerated NP tissue was characterized with increased extracellular matrix (ECM) stiffness,^13^ mainly caused by dysfunction of anabolism and catabolism of collagen, proteoglycans, and complex glycoproteins in NPCs, contributing to normal matrix disintegration and fibrous matrix formation.^14,15^ Recent studies revealed that increased matrix stiffness led to aberrant glycolysis in epithelial cells and tumors via activation of mechanotransduction signaling pathways.^16,17^ However, whether increased matrix stiffness in NP tissues can modulate glycolysis during IVDD progression is unknown.

Cellular mechanotransduction induced by ECM stiffness is sensed by integrin-focal adhesion complexes on the cell membrane and transmitted into the intracellular, and cytoplasmic mechanical cues can relay to the nucleus to activate downstream pathways.^18^ Myocardin-related transcription factor-A (MRTF-A) as an important mediator in stiffness signaling mechanotransduction that mainly responds to cytoskeletal remodeling signals.^18,19^ As a potent coactivator of serum-response factor (SRF) transcription factor, MRTF-A binds to serum-response elements (SREs) to regulate transcriptions that affect cell differentiation,^19–21^ ECM remodeling,^22,23^ and fibrosis.^18,24^ Matrix stiffness mechanotransduction in epithelial and tumor cells altered glycolysis, reflecting the connection between ECM stiffness and glucose metabolism.^16,17^ Given that role of MRTF-A in matrix stiffness mechanotransduction and the potential link between matrix stiffness and glycolysis, we hypothesize that MRTF-A is a regulator between ECM stiffness and glycolysis.

In this study, we investigated the effect and mechanism of ECM stiffness on NPC glycolysis and identified the key regulator as a potential therapeutic target for IVDD. Our findings revealed that increased matrix stiffness suppressed glycolysis with a reduction in glycolytic enzyme expression in degenerated NP tissues and NPCs cultured in rigid conditions. MRTF-A was upregulated during IVDD progression, and MRTFA overexpression decreased glycolysis products including lactic acid, pyruvic acid, and ATP production with GC-MS analysis. Furthermore, rigid substrates and MRTF-A overexpression inhibited Kidins220 expression and AMPK activation in NPCs, and MRTF-A ablation partially increased glycolysis by promoting Kidins220 expression and AMPK phosphorylation. Finally, we observed a recovery of NP tissue degeneration and expression of glycolytic enzymes and downstream pathways, including AMPK and Kidins220, after treatment with CCG, a MRTF-A inhibitor. Our data demonstrate that MRTF-A is a critical regulator that responds to increased matrix stiffness in IVDD, and MRTF-A activation reduces NPC glycolysis by down-regulating Kidins220 and inhibiting AMPK phosphorylation.

## Results

### Increased matrix stiffness inhibits glycolysis in NPCs

**Fig. 1:**
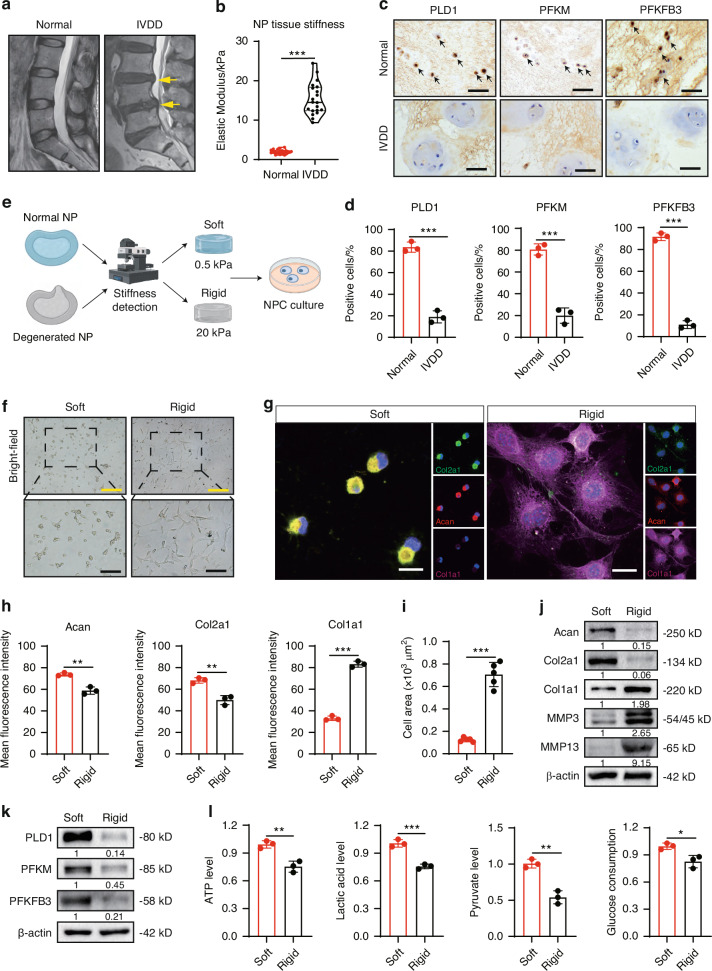
Matrix stiffness affects the anabolism and glycolysis of NPCs. **a** T2-weighted MRI images of normal and IVDD group IVDs; the yellow arrows indicate the degenerative NP tissues. **b** The stiffness analysis of NP tissues in the normal (*n* = 20) and IVDD groups (*n* = 20). **c**, **d** The IHC images of NP tissues and their quantification (black arrows indicate representative positive cells). **e** The flowchart for the preparation of substrates for the culture of NPC based on the stiffness of NP tissues. **f** Bright field images of NPCs cultured on soft or rigid substrates. **g**, **h** The IF staining (Col2a1, Acan, and Col1a1) of NPCs and quantification. **i** Cell area analysis of NPCs cultured on soft or rigid substrates. **j**, **k** The levels of Acan, Col2a1, Col1a1, MMP3, MMP13, PLD1, PFKM and PFKFB3 in NPCs cultured on soft or rigid substrates for 24 h. **l** The relative levels of ATP, lactic acid, and pyruvate production and glucose consumption in culture supernatant collected from NPCs cultured on soft or rigid substrates for 24 h. Yellow scale bar = 400 μm, black scale bar = 200 μm, and white scale bar = 20 μm. *P < 0.05, **P < 0.01, ***P < 0.001

In line with our prior study,^25^ the T2-weighted magnetic resonance imaging (MRI) showed different signal intensities in normal and degenerated IVDs (Fig. 1a), and the degenerated NP tissue displayed markedly increased stiffness (Fig. 1b). Specifically, the mean stiffness of NP tissues in IVDD patients was 15.34 ± 4.05 kPa, substantially stiffer than that of the normal group (1.972 ± 0.55 kPa). Numerous studies have demonstrated that increased ECM stiffness regulated cellular glucose metabolism in tumors.^17,26,27^ To explore the changes of glycolysis in degenerated NP tissues, we detected three critical enzymes involved in glycolysis and glucose uptake processes. Immunohistochemistry (IHC) staining showed phospholipase D1 (PLD1) and phosphofructokinase-1 (PFKM) were decreased to 20% in degenerated NP tissues (Fig. 1c, d). Notably, 6-phosphofructo-2-kinase/fructose-2,6-biphosphatase 3 (PFKFB3), which serves as a potent allosteric activator of the rate-controlling glycolytic enzyme PFKM to sustain glycolysis,^28^ was approximately seven-fold lower than that in normal group (Fig. 1c, d).

To mimic the matrix stiffness in normal and degenerated NP tissues, we established soft (0.5 kPa) and rigid (20 kPa) polyacrylamide hydrogel substrates to culture NPCs based on differences in NP tissue stiffness measured by atomic force microscope (Fig. 1e). NPCs cultured on soft hydrogel showed circularity, whereas they presented polygonal and vacuole-free shapes on rigid substrates (Fig. 1f). The major chondrocyte markers of NP cells are type II collagen (Col2a1) and aggrecan (Acan), whereas the fibrous marker is type I collagen (Col1a1).^29,30^ Immunofluorescence (IF) staining confirmed that Col2a1 and Acan predominantly accumulated in primary NPCs from rats cultured on soft substrates, whereas rigid substrates dramatically upregulated Col1a1 expression and increased cell area (Fig. 1g–i). Similarly, compared to soft condition, rigid substrate decreased expression of Acan and Col2a1 while increasing Col1a1, MMP3, and MMP13 levels in NPCs (Fig. 1j), suggesting degenerated phenotype of NPCs with increased matrix stiffness. Consistent with degenerated NP tissues, rigid substrate decreased PLD1, PFKM, and PFKFB3 expression in NPCs (Fig. 1k). Moreover, rigid substrate decreased levels of ATP, lactic acid, and pyruvate and suppressed glucose consumption in NPCs (Fig. 1l). Collectively, these results indicated that increased matrix stiffness impaired glycolysis process of NPCs with reduction of glycolytic enzyme expression.

### MRTF-A activation by matrix stiffness promotes NPC degeneration

**Fig. 2:**
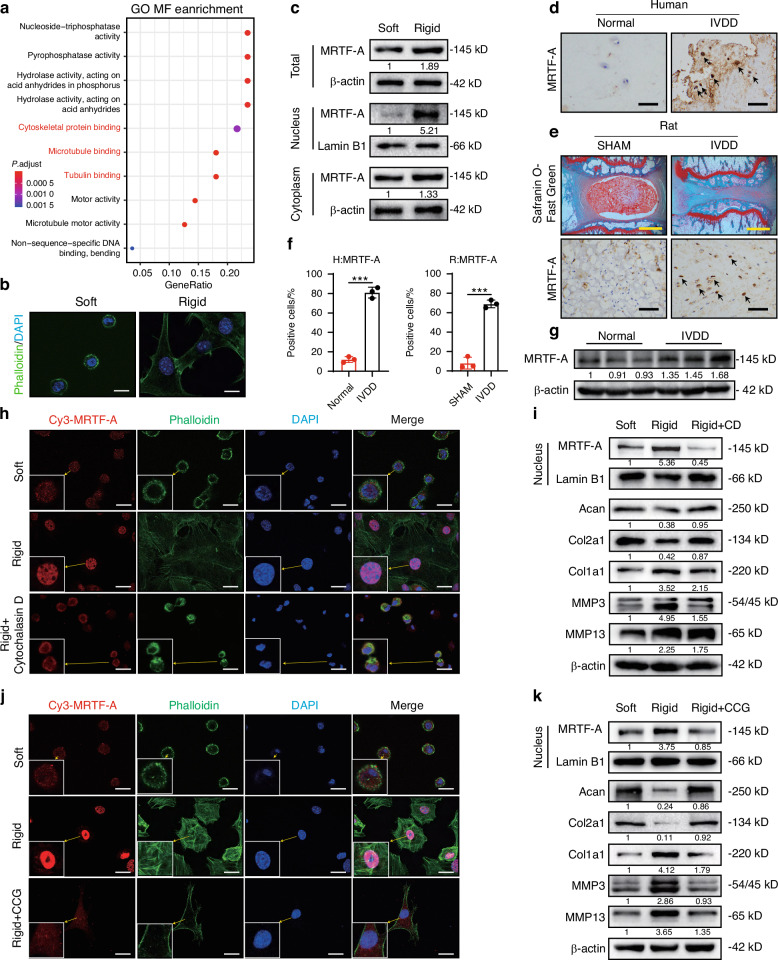
The effects of MRTF-A in NPCs. **a** The Go enrichment analysis (MF) in NPCs cultured on soft or rigid substrates (top 10). **b** The phalloidin staining of NPCs cultured on soft or rigid substrates. **c** The level of MRTF-A in the nucleus and cytoplasm of NPCs cultured on soft or rigid substrates for 24 h. **d** The MRTF-A IHC images of NP tissues from normal and IVDD human (black arrows indicate representative positive cells). **e** The safranin O-fast green staining and MRTF-A IHC images of NP tissues from SHAM and IVDD rat models (black arrows indicate representative positive cells). **f** Quantification of MRTF-A IHC images. **g** The levels of MRTF-A in normal and IVDD group NP tissues. **h** The IF images of MRTF-A (Cy3) and phalloidin (FITC) staining of NPCs treated with CCG for 24 h under rigid substrate condition. **i** The levels of MRTF-A (nucleus), Acan, Col2a1, Col1a1, MMP3 and MMP13 in NPCs treated with 50 μmol/L CCG for 24 h under ridig substrate condition. **j** The IF images of MRTF-A (Cy3) and phalloidin (FITC) staining of NPCs treated with CD for 24 h under rigid substrate condition. **k** The levels of MRTF-A, Acan, Col2a1, Col1a1, MMP3 and MMP13 in NPCs treated with CD for 24 h under rigid substrate condition. Yellow scale bar = 1 000 μm, black scale bar = 200 μm, and white scale bar = 20 μm. ***P < 0.001

To investigate the mechanism of matrix stiffness affecting glycolysis in NPCs, we performed RNA sequencing with NPCs cultured on soft and rigid substrates. The genes upregulated and downregulated > 1.5 folds with *P*-value < 0.05 were deemed to be significantly differentially expressed (Fig. S1). Volcano plot results showed that rigid culture conditions upregulated 435 genes and downregulated 797 genes in NPC (Fig. S1c, d). Further GO enrichment analysis revealed that the rigid substrate altered cytoskeleton-related biological processes, including cytoskeletal protein binding, microtubule binding, and tubulin binding (Fig. 2a). Subsequently, phalloidin staining showed that rigid substrate promoted NPC cytoskeleton remodeling (Fig. 2b). MRTFA, a mechanosensitive transcriptional coactivator in matrix stiffness mechanotransduction mainly responding to cytoskeleton remodeling,^18,19^ was increased and translocated to the nucleus under rigid substrate condition (Fig. 2c). Consistent with NPCs, IHC staining also showed that MRTF-A was six- and five-fold upregulated in the NP tissues of IVDD patients and rat model, respectively (Fig. 2d–f and Fig. S2a). Similarly, western blotting analysis showed MRTF-A was markedly increased in degenerated NP tissue (Fig. 2g). These results indicated that NP tissue degeneration may be intimately linked to cytoskeletal remodeling and MRTF-A activation.

To validate the role of cytoskeletal remodeling in NPC matrix stiffness mechanotransduction, Cytochalasin D (CD), a potent inhibitor of actin polymerization,^31^ was used to inhibit the cytoskeletal remodeling process. As shown in Fig. 2h, rigid substrates not only enhanced F-actin polymerization in NPCs and increased cytoskeletal area, but also stimulated cytoplasmic MRTF-A nuclear translocation. However, CD treatment effectively inhibited cytoskeletal remodeling and MRTF-A nuclear translocation (Fig. 2h). Notably, CD treatment partially rescued the rigid matrix-induced NPC degenerative phenotype (Fig. 2i). Therefore, the effect of matrix stiffness on NPC may be mediated by cytoskeletal remodeling-associated MRTF-A activation.

To further demonstrate the role of MRTF-A in regulating NPC function, we examined the expression of ECM components. Overexpression of MRTF-A in NPCs resulted in increased expression of the Col1a1 and catabolism markers (MMP3 and MMP13) and decreased expression of chondroid elements, including Col2a1 and Acan (Fig. S2b). Inhibition of MRTF-A translocation to nuclear with CCG, a MRTF-A inhibitor, in NPCs on rigid substrate, and 50 μmol/L concentration was used in subsequent experiments (Fig. S2c). Immunofluorescence staining also displayed that CCG suppressed MRTF-A nuclear translocation, but had no effect on the cytoskeleton (Fig. 2j). Importantly, CCG treatment restored the expression of Acan and Col2a1 and decreased Col1a1, MMP3, and MMP13 levels under rigid substrate, reflecting that inhibition of MRTF-A partially rescued the NPC dysfunction induced by increased matrix stiffness (Fig. 2k). Together, these data suggested that MRTF-A was upregulated during IVDD progression, and its activation induced by increased stiffness exacerbated NPC metabolic dysfunction.

### MRTF-A reduces glycolysis via inhibiting the AMPK pathway in NPCs

**Fig. 3:**
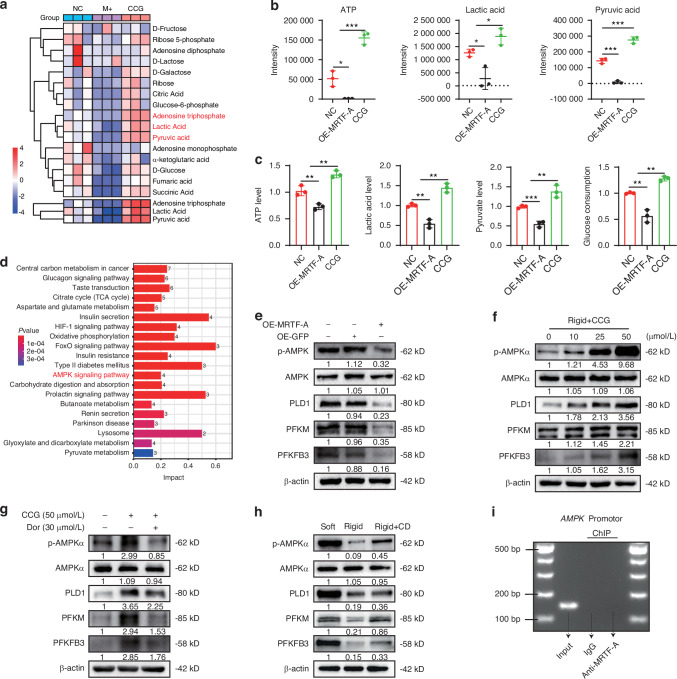
MRTF-A regulates glycolysis in NPCs via the AMPK pathway. **a** Heat map of metabolites that were significantly differentially expressed (*P* < 0.05). **b** The intensity quantification of ATP, lactic acid, and pyruvic acid in GC-MS analysis results. **c** The relative levels of ATP, lactic acid, pyruvate production and glucose consumption in culture supernatant collected from NPCs treated with OE-MRTF-A or CCG. **d** KEGG enrichment analysis of GC-MS analysis results (top 10). **e** The levels of p-AMPKα, AMPKα, PLD1, PFKM and PFKFB3 in NPCs treated with OE-GFP or OE-MRTF-A for 24 h. **f** The levels of p-AMPKα, AMPKα, PLD1, PFKM and PFKFB3 in NPCs treated with 0, 10, 25, and 50 μmol/L CCG for 24 h under ridig substrate condition. **g** The levels of p-AMPKα, AMPKα, PLD1, PFKM and PFKFB3 in NPCs were treated with 50 μmol/L CCG alone or combined with 30 μmol/L Dor for 24 h under ridig substrate condition. **h** The levels of p-AMPKα, AMPKα, PLD1, PFKM and PFKFB3 in NPCs were treated with CD for 24 h. **i** The PCR image of AMPK promoter region (MRTF-A antibody was used for immunoprecipitation, with normal IgG as the negative control and input as the loading control). *P < 0.05, **P < 0.01, ***P < 0.001

Considering that increased matrix stiffness resulted in insufficient glycolysis and MRTF-A was an effector that responded to increased matrix stiffness in NP tissue, we further explored whether the activation of MRTF-A can regulate glycolysis in NPCs. After treatment with OE-MRTF-A and CCG, the metabolites in NPCs were evaluated using GC-MS analysis. Significantly differentially expressed metabolites were identified, mainly involving glucose metabolism (Fig. 3a, b and Fig. S3a, b). MRTF-A overexpression significantly reduced glycolysis products, including lactic acid, pyruvic acid, and ATP production, whereas CCG treatment increased the levels of these glycolysis products (Fig. 3b). Similarly, the determination of glycolytic indicators in cell supernatant displayed that OE-MRTF-A treatment declined levels of ATP, lactic acid, and pyruvate and reduced glucose consumption, and CCG reversed this trend (Fig. 3c). The KEGG enrichment analysis further showed that changes in glycolysis function were linked to several metabolic pathways, such as the AMPK signaling pathway, carbohydrate digestion and absorption, and pyruvate metabolism (Fig. 3d and Fig. S3b). Among these, the AMPK pathway was also found to correlate with changes in ECM stiffness in the KEGG enrichment analysis of NPC RNA sequencing (Fig. S3c). The previous study demonstrated that AMPK pathway modulated glycolysis through the PFKM and PFKFB3 enzymes, as well as glucose absorption via PLD1.^32^ Notably, rigid substrates inhibited AMPK phosphorylation and downstream PLD1, PFKM, and PFKFB3 expression, whereas GSK, an AMPK agonist, treatment partially rescued the glycolytic function of NPC via promoting AMPK phosphorylation (Figs. S4a, b and S5a, b). These data revealed that matrix stiffness reduced NPC glycolysis primarily through inhibition of the AMPK pathway.

Subsequently, overexpression of MRTF-A in NPCs inhibited AMPK phosphorylation and diminished the expression of PLD1, PFKM, and PFKFB3 (Fig. 3e and Fig. S5c). On the contrary, CCG activated AMPK phosphorylation in NPCs in a dose-dependent manner, thereby upregulating PLD1, PFKM, and PFKFB3 expression (Fig. 3f and Fig. S5d). However, Dor, an AMPK inhibitor, decreased AMPK phosphorylation and glycolysis enzyme upregulation caused by CCG treatment (Fig. 3g, Figs. S4c, d and S5e). In addition, inhibition of cytoskeletal remodeling also maintained AMPK phosphorylation, suggesting that cytoskeletal remodeling and subsequent MRTF-A activation were associated with impaired glycolysis (Fig. 3h and Fig. S5f). Although these results demonstrated a regulatory relationship between MRTF-A and AMPK, the ChIP results suggested that MRTF-A cannot directly regulate the transcription process of AMPK (Fig. 3i).

### MRTF-A inhibits AMPK phosphorylation and reduces glycolysis by decreasing Kidins220 expression

**Fig. 4:**
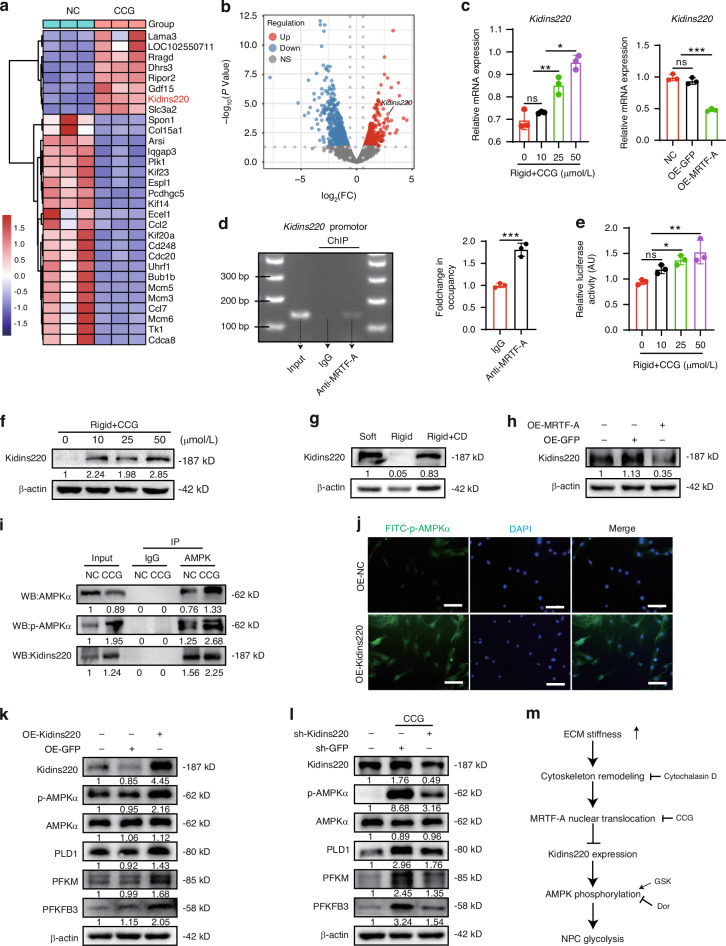
MRTF-A inhibits Kidins220 expression, limiting AMPK phosphorylation and glycolysis. **a** Heat map of top 30 significantly differentially expressed genes in NPCs after CCG treatment. **b** Volcano plot of significantly differentially expressed genes (Foldchange ≥ 1.5, *P* < 0.05). **c** The mRNA level of *Kidins220* in NPCs were treated with CCG or OE-MRTF-A. **d** The PCR image of Kidins220 promoter region (MRTF-A antibody was used for immunoprecipitation, with normal IgG as the negative control and input as the loading control). **e** Relative luciferase activity in HEK 293T cells treated with CCG for 24 h. **f**–**h** The levels of Kidins220 in NPCs treated with different concentrations of CCG, CD or OE-MRTF-A. **i** AMPK was immunoprecipitated from NPCs after CCG treatment, and the presence of AMPK, p-AMPK, and Kidins220 in the immunoprecipitates was evaluated. **j** IF images of p-AMPK in NPCs transfected with OE-Kidins220 for 24 h. **k** The levels of Kidins220, p-AMPK, AMPK, PLD1, PFKM, and PFKFB3 in NPCs transfected with OE-GFP or OE-Kidins220 for 24 h. **l** The levels of Kidins220, p-AMPK, AMPK, PLD1, PFKM, and PFKFB3 in NPCs transfected with sh-Kidins220 and treated with CCG for 24 h. **m** Schematic diagram of multiple treatments. White scale bar = 200 μm. *P < 0.05, **P < 0.01, ***P < 0.001

To explore the specific mechanism by which MRTF-A regulates AMPK phosphorylation, RNA sequencing data was analyzed and mapped the differential expression genes in NPCs treated with CCG. We identified 946 significantly differentially expressed genes and listed the top 30 genes (Fig. 4a). Among these genes, *Kidins220*, which is associated with potassium channel protein expression and ATP production,^33^ was strikingly increased in CCG-treated NPCs (Fig. 4b). Gene expression of *Kidins220* was demonstrated to be negatively regulated by activity of MRTF-A (Fig. 4c). Furthermore, ChIP analysis revealed that MRTF-A was bound to the promoter of *Kidins220* (Fig. 4d), while luciferase analysis showed that CCG treatment directly promoted *Kidins220* transcription (Fig. 4e). CCG and CD treatment promoted Kidins220 protein level under rigid substrates, while MRTF-A overexpression reduced its expression (Fig. 4f–h).

To investigate the regulatory mechanism between Kidins220 and AMPK, endogenous Co-IP showed that Kidins220 interacted with AMPK, and CCG augmented the interaction between Kidins220 and AMPK (Fig. 4i). Moreover, Kidins220 overexpression promoted AMPK phosphorylation and increased glycolysis enzyme expression, whereas Kidins220 knockdown partially reduced AMPK phosphorylation and glycolysis induced by CCG treatment (Fig. 4j–l and Fig. S5g–i). Taken together, activated MRTF-A inhibited Kidins220 expression, and thereby inhibiting AMPK phosphorylation and glycolysis process (Fig. 4m).

### MRTF-A overexpression in NP tissue accelerates IVDD progression

**Fig. 5:**
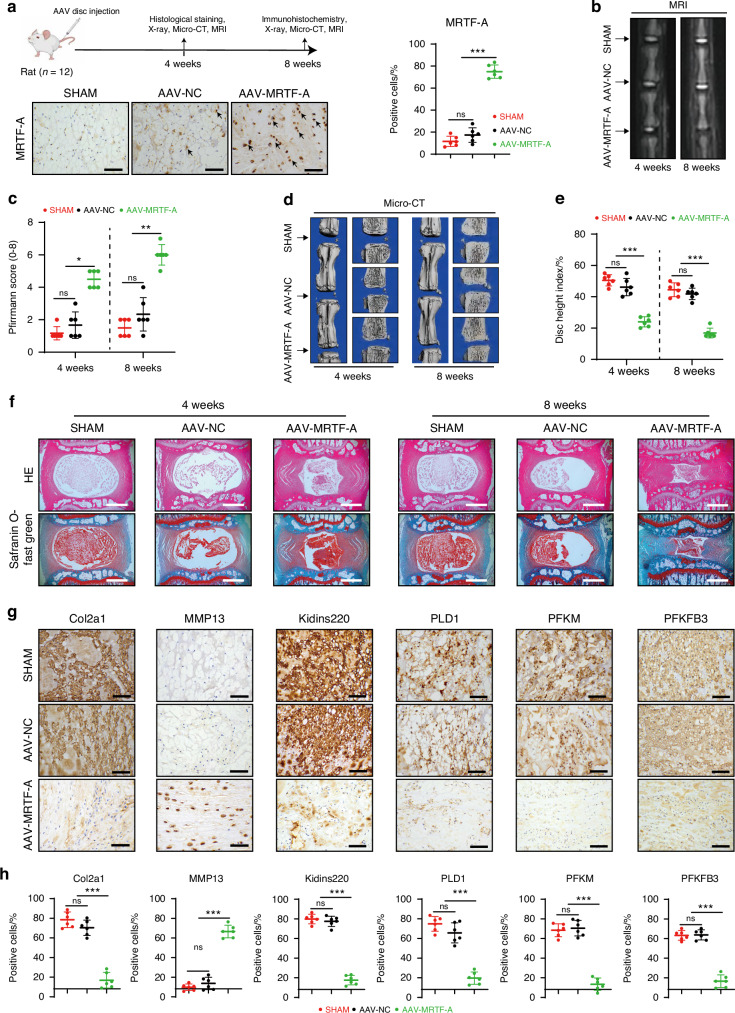
MRTF-A overexpression promotes the IVDD progression. **a** Schematic diagram of the process of rats receiving AAV-MRTF-A injections (black arrows indicate representative positive cells). **b**, **c** The MRI images of rat discs and Pfirrmann score quantification (0–8). **d**, **e** CT three-dimensional images of rat intervertebral discs and disc height index quantification. **f** The histological staining images of intervertebral discs at 4 and 8 weeks after treatment. **g**, **h** The IHC images of intervertebral discs at 8 weeks after treatment and its quantitative analyses. Black scale bar = 200 μm and white scale bar = 1 000 μm. *P < 0.05, **P < 0.01, ***P < 0.001

To investigate the role of MRTF-A in IVDD progression, we subsequently injected AAV-MRTF-A into the NP tissue of rats. As shown in Fig. 5a and Fig. S6a, more than four-fold increased expression of MRTF-A was observed in NP tissues following AAV-MRTF-A injection, indicating stable upregulation of MRTF-A in NP tissues. The MRI analysis showed lower signal density and increased Pfirrmann grades of the IVDs treated with AAV-MRTF-A compared to SHAM or AAV-NC injection (Fig. 5b, c). X-ray scan and micro-CT 3D reconstruction of IVDs showed reduction of IVD height and increased osteophytes in AAV-MRTF-A-treated disc (Fig. 5d, e and Fig. S6b). HE and Safranin O/Fast Green staining displayed that MRTF-A overexpression led to NPC loss, matrix collapse, and annulus fibrosus fissures (Fig. 5f and Fig. S6c). Notably, AAV-MRTF-A treatment not only increased MMP13 and decreased Col2a1 expression in NP tissues, but also reduced glycolysis enzymes and Kidins220 expression (Fig. 5g, h and Fig. S6d–i). These results revealed that overexpression of MRTF-A was involved in the onset and development of IVDD.

### MRTF-A inhibition partially mitigates IVDD progression

**Fig. 6:**
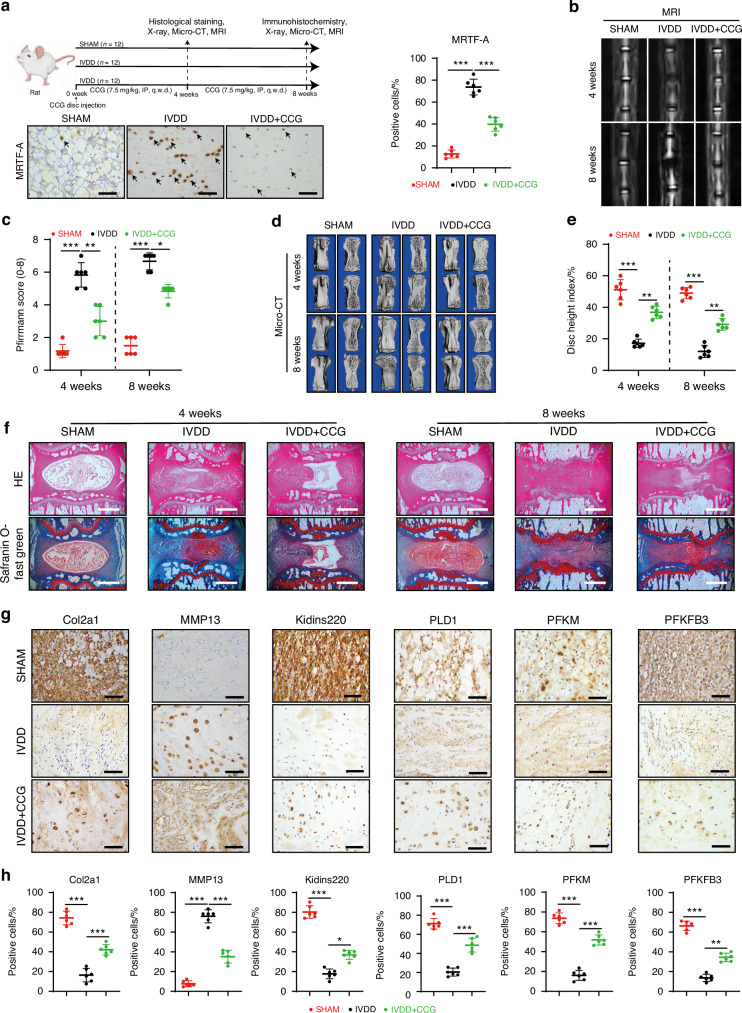
Inhibition of MRTF-A alleviates the IVDD progression. **a** Schematic diagram of the process of rats receiving puncture surgery and CCG treatment (black arrows indicate representative positive cells). **b**, **c** The MRI images of rat discs and Pfirrmann score quantification (0–8). **d**, **e** CT three-dimensional images of rat intervertebral discs and disc height index quantification. **f** The histological staining images of intervertebral discs at 4 and 8 weeks after treatment. **g**, **h** The IHC images of intervertebral discs at 8 weeks after treatment and its quantitative analyses. Black scale bar = 200 μm and white scale bar = 1 000 μm. *P < 0.05, **P < 0.01, ***P < 0.001

To further explore the potential of MRTF-A as a therapeutic target for IVDD, we generated an IVDD rat model to administrate with MRTF-A inhibitor CCG (Fig. 6a and Fig. S7a). The imaging evaluation of degenerated IVDs induced by puncture was characterized by lower disc height and T2 signal density as well as more structural damage than the SHAM group, and CCG partially rescued collapsed disc structure and phenotype (Fig. 6b–e and Fig. S7b). At the histological level, CCG administration alleviated loss of NP tissue and disc morphological destruction in IVDD group (Fig. 6f and Fig. S7c). Compared with IVDD group, CCG treatment diminished MRTF-A and MMP13 levels and increased Col2a1 expression in the NP tissues, indicating that MRTF-A expression was related to NP tissue degeneration (Fig. 6g, h and Fig. S7d–i). Moreover, CCG treatment partially increased Kidins220 expression and restored PLD1, PFKM, and PFKFB3 expression. Together, inhibiting MRTF-A partially rescued the anabolism and glycolysis of NPCs and alleviated the progression of IVDD.

## Discussion

**Fig. 7:**
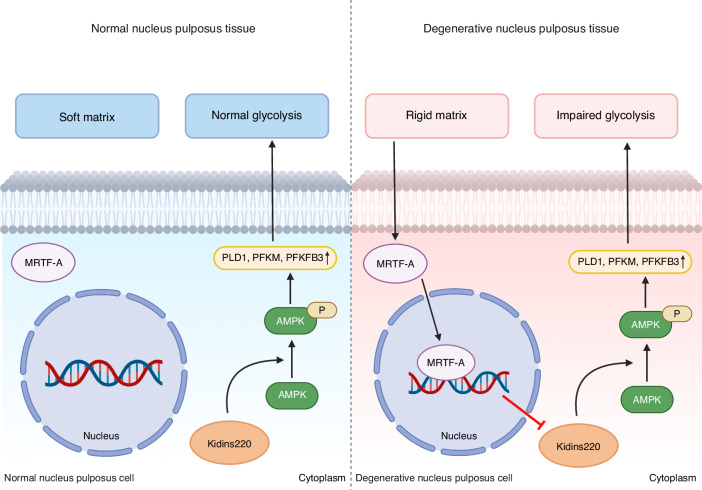
Schematic diagram depicting the mechanism of the rigid matrix regulating cell glycolysis in degenerated NPCs. In degenerated NP tissue, MRTF-A was activated by increased matrix stiffness and transferred into the nucleus, thus reducing Kidins220 expression and inhibiting AMPK phosphorylation

Aberrant matrix stiffness in many tissues can contribute to metabolic dysfunctions, including glucose,^16,17,34^ lipid,^35,36^ and protein metabolism.^37^ The glycolysis process is the main source of ATP production in NPCs, providing energy for survival and ECM formation.^10,11^ IVDD progression is accompanied with increased stiffness of NP tissue, which can be sensed by NPCs and hence regulate protein expression and signal transduction.^13,25^ However, how matrix stiffness reduced NPC glycolysis was not yet fully elucidated in NPCs. This study demonstrated a mechanistic link between matrix stiffness and impaired glycolysis in IVDD progression and identified MRTF-A as a critical factor in mechanotransductive process. Increased matrix stiffness in degenerative NP tissue led to NPC dysfunction and weakened glycolysis via MRTF-A activation. On the contrary, inhibition of MRTF-A with CCG partially rescued NPC dysfunction and glycolysis in vitro and improved NP tissue degeneration in rat IVDD model. Mechanistically, MRTF-A reduced the glycolysis of NPC mainly by diminishing the expression of Kidins220 and inhibiting AMPK phosphorylation. Together, our findings revealed that enhanced matrix stiffness triggered nuclear translocation of MRTF-A, which reduced Kidins220 transcription and expression, thereby suppressing AMPK activation and glycolysis (Fig. 7).

Augmented matrix stiffness is typically associated with disease progression.^38,39^ Likewise, matrix-stiff lesions were also discovered in the deteriorated human NP tissue, and rigid substrate culture conditions contributed to NPC malfunction.^25^ Numerous studies have demonstrated that several mechanosensitive proteins activated by stiffness can affect NPC function and promote IVDD progression, including YAP/TAZ transcription factor,^13,25^ integrin β1 receptor,^40^ and Piezo1 ion channel.^41,42^ These evidences demonstrate the interaction between ECM stiffness properties and pathogenesis during IVDD progression. MRTF-A is an essential nuclear factor activated in response to cytoskeletal remodeling that can regulate transcription processes as well as engage in various disease progressions related to matrix stiffness.^23,43,44^ Herein, we found that rigid substrates also activated MRTF-A in NPCs, resulting in NPC degeneration and IVDD progression. On the contrary, suppressing MRTF-A mitigated the negative effects caused by matrix stiffness. These evidences emphasize the link between ECM stiffness and IVDD progression even more, and MRTF-A exerts a critical function in this process.

MRTF-A is a transcriptional coactivator that physically associates with the SRF and synergistically activates transcription, thus regulating a variety of biological processes, such as proliferation, differentiation, apoptosis, and fibrosis.^45,46^ It represents a central relay in stiffness mechanotransduction and is controlled by the actin cytoskeleton to activate nuclear translocation and regulate transcription.^18,45^ Previous studies widely regarded MRTF-A as directing a subset of fibrosis target genes, such as *Col1a1*, *α-SMA*, and *vimentin*, thus regulating fibroblast differentiation,^19,24^ tumor growth and invasion,^23,47^ as well as tissue fibrosis.^24,44,48^ Notably, existing studies revealed that MRTF-A activation is associated with the fibrotic phenotype of NPC in IVDD progression,^49–52^ but the specific mechanism is unclear. In this study, GC-MS analysis revealed that MRTF-A activation caused impaired glycolysis, which might explain why stiffness affects chondrogenic phenotype and glycolysis in NPCs. Furthermore, CCG targeting MRTF-A has been widely investigated as a promising antifibrotic agent,^53^ and we optimized the CCG intervention conditions to improve efficacy against IVDD in the present study. On the one hand, we applied a novel second-generation inhibitor, CCG-203971, instead of the first-generation CCG-1423,^50,52^ which helps to minimize toxic side effects and improve therapeutic specificity.^44^ On the other hand, CCG-203971 is characterized by poor metabolic stability and a short half-life, making it difficult to sustain drug concentration in NP tissue.^53^ As a result, in addition to direct 2 µL CCG injections in NP tissues (2.5 µg/µL) based on prior research,^50^ intraperitoneal administration of CCG (concentration: 2.5 mg/mL, dosing: 7.5 mg/kg) was performed weekly to maintain drug concentrations until the endpoint of the experiment. The results suggested that this CCG treatment strategy could effectively inhibit MRTF-A activity and ameliorate NP tissue degeneration without obvious side effects during the experimental duration.

The matrix stiffness can affect the glycolysis function within various cells by regulating the mechanotransduction process, including key proteins,^26,27^ transcription factors,^16,17,34^ and cytoskeleton architecture.^54^ Recent studies reported that matrix stiffness enhances glycolysis via YAP activation in cancer cells and carcinoma-associated fibroblasts.^17,34^ Our findings revealed that NPCs were predominantly affected by cytoskeletal remodeling processes on rigid substrates and that cytoskeletal inhibitor CD treatment partially rescued glycolytic functions. However, MRTF-A also serves as a mechanosensitive transcriptional coactivator regulated by the cytoskeleton; its effect on glycolysis is still unknown. Although MRTF-A can affect NPC phenotype and shape,^49^ it is still unclear how MRTF-A regulates the glycolysis cycle in NPCs. According to GC-MS analysis, we discovered that MRTF-A activation reduced glycolysis via inhibiting the AMPK pathway. AMPK is intimately involved in the process of glucose metabolism, as it not only regulates PLD1 to improve cell glucose uptake,^55^ but also increases flux along the glycolytic path by phosphorylating PFKFB3 and affecting PFKM.^28^ Furthermore, AMPK plays a dual role in the pathogenesis of IVDD: on the one hand, AMPK protects NPCs by promoting autophagy,^56^ reducing inflammation,^57^ resisting oxidative stress and senescence;^58^ on the other hand, AMPK can inhibit cell proliferation and delay cell cycle progression by inhibiting the mTOR pathway.^59,60^ Herein, AMPK, limited by MRTF-A, served as a key target for regulating glycolysis to maintain energy in NPCs.

Recent research suggests that AMPK has been shown to phosphorylate cofilin in fibroblasts, resulting in cytoskeletal remodeling and MRTF-A activation.^61^ However, our results indicated that activation of MRTF-A in NPCs reduced the phosphorylation of AMPK, and whether AMPK could affect MRTF-A remained to be explored. To affect AMPK phosphorylation in the cytoplasm, intranuclear MRTF-A reduced the transcription and expression of Kidins220 based on RNA sequencing, ChIP and a double luciferase assay. Kidins220 is a conserved membrane protein that interacts with the microtubule and actin cytoskeleton and activates distinct signaling cascades.^62,63^ At present, numerous studies indicated that Kidins220 is primarily involved in the potassium channel protein expression and ATP production,^33^ the development of the nervous and vascular systems, and the survival and differentiation of neurons.^64,65^ Our current work discovered that MRTF-A in the nucleus reduced the transcription and expression of Kidins220, consequently inhibiting AMPK phosphorylation and glycolysis processes. However, further investigation into the involvement of Kidins220 in the IVDD progression is still required.

Although our study provided detailed insights into the interactions between matrix stiffness and glycolysis, there are some limitations for subsequent research and clinical conversion. Firstly, gene therapy and CCG treatment targeting MRTF-A presently show potential to rescue existing NPC function, but it is difficult to eliminate the aberrant ECM component deposition and reduce the stiffness of NP tissues. It implies that these treatments may be more appropriate for patients with early-stage IVDD to prevent further NP tissue degeneration; however, subsequent clinical application is still subject to the potential challenges of delivery method and clinical safety. Second, there is no doubt that appropriate glycolysis flux is essential for NPCs, and excessive or insufficient glycolysis can have adverse effects.^12,66,67^ However, how to define appropriate standards in cells or tissues has always been controversial. It may be challenging to solve this problem just by identifying glycolytic enzymes or synthetic metabolic indicators in cells. Third, the analysis of matrix stiffness mechanotransduction in this experiment began with the activation of MRTF-A, which may have neglected the effect of upstream processes on Kidins220 and AMPK. In particular, both Kidins220 and AMPK have been reported to be associated with cytoskeletal remodeling.^61,62^

Despite these limitations, our research revealed an association between matrix stiffness and glycolysis in the course of IVDD and highlighted the crucial role of MRTF-A, triggered by aberrant stiffness signals, that leads to impaired glycolysis and IVDD progression by inhibiting Kidins220-AMPK signaling. The findings provided insights into the underlying mechanisms of impaired glycolysis by which the Kidins220-AMPK pathway is involved in the activation of MRTF-A. As a result, we suggested that MRTF-A may serve as a significant target for restoring glycolysis and delaying the progression of IVDD induced by ECM stiffness.

## Materials and methods

### Experimental reagents and animals

The CCG-203971 (CCG, HY-108361), Cytochalasin D (CD, HY-N6682), GSK621 (GSK, HY-100548), Dorsomorphin (Dor, HY-13418A), and Phalloidin (HY-P0028) were purchased from MedChemExpress (NJ, USA). The MRTF-A overexpressed adenovirus (OE-MRTF-A) and adeno-associated virus (AAV-MRTF-A), Kidins220 overexpressed (OE-Kidins220), and knockdown adenovirus (sh-Kidins220), as well as their negative control vector were constructed by Vigene Biosciences (Shandong, China).

The male Sprague-Dawley rats (6–8 weeks, 200 ± 20 g) were obtained from Vital River Laboratories (Beijing, China). The rats were fed in specific pathogen free cages and allowed to move and obtain autoclaved food and water *ad libitum*. The raising conditions were kept at at 24 ± 1 °C, 55% ± 10% humidity, and 12 h light/dark cycles. All animal experiments conformed to the stipulations in the World Medical Association Helsinki Declaration and were approved by the Ethics Committee of Huazhong University of Science and Technology (TJH-202107013).

### NPC isolation, culture and treatment

NP tissues were obtained from the tail intervertebral disc of rats and digested with 0.25% trypsin for 30 min and type II collagenase for 3 h at 37 °C. Subsequently, NPCs were collected and cultured in DMEM/F12 medium (Gbico, NY, USA) supplemented with 10% fetal bovine serum (Gbico) and 1% penicillin/streptomycin (Gibco) in an incubator (37 °C, 5% CO_2_).

For adenovirus transfection, NPCs were incubated with OE-MRTF-A (MOI = 100), OE-Kidins220 (MOI = 100), or sh-Kidins220 (MOI = 100) in medium without antibiotics. The infection medium was replaced by growth medium after 12 h. In addition, the experimental concentrations and durations of CCG (50 μmol/L, 24 h), GSK (60 μmol/L, 3 h), and Dor (30 μmol/L, 24 h) in NPC were determined through gradient experiments.

### Human NP tissue collection and stiffness detection

All patients were selected from the inpatients that underwent lumbar discectomy in our facility. NP tissue acquisition was approved by patients and the Ethics Committee of Huazhong University of Science and Technology (TJ-IRB20220609). Normal group NP tissues were obtained from patients who needed lumbar discectomy but had a Pfirrmann score ≤ II (*n* = 20); IVDD group NP tissues were obtained from patients who needed lumbar discectomy but had a Pfirrmann score ≥ III (*n* = 20). NP tissues were prepared as a standard sample, and stiffness detection was performed by an atomic force microscope (Agilent-5500, CA, USA).

### Polyacrylamide hydrogel preparation

Polyacrylamide hydrogels were prepared, and the Young’s modulus of polyacrylamide hydrogels was measured as previously described.^23^ Briefly, hydrogel stiffness was varied by changing the relative concentrations of acrylamide (Sigma-Aldrich, Wisconsin, USA) and bis-acrylamide (Sigma-Aldrich), and then adding 10% ammonium persulfate (Sigma-Aldrich) and 1% TEMED (Sigma-Aldrich) for polymerization. Bovine type II collagen (BD Biosciences, NJ, USA) was covalently bound to substrates using the heterobifunctional cross-linker sulfo-SANPAH (Sigma-Aldrich). NPCs were cultured on different stiffness hydrogels for 24 h and collected for subsequent experiments.

### Western blot analysis

NP tissue and cell proteins were isolated using RIPA lysis (Boster, Wuhan, China), and their concentrations were determined by the BCA Assay Kit (Boster). The protein samples (20 µg) were separated using SDS-PAGE gel electrophoresis and electrotransferred to PVDF membrane (Millipore, MA, USA). Subsequently, membranes were blocked with 5% BSA (BioFroxx, Germany) for 1 h and incubated with primary antibodies (Table S1) overnight at 4 °C. The next day, the secondary antibodies (Boster) were used to immunoblot the membranes for 1 h, and SuperSignal West Pico PLUS Chemiluminescent Substrate (Thermo Fisher Scientific, MA, USA) was used to visualize the blot images. The ChemiDoc XRS Imaging System (Bio-Rad, CA, USA) was used to detect and analyze the blot signals.

### Co-Immunoprecipitation (Co-IP)

NPCs were lysed in immunoprecipitation lysis buffer for 30 min, and the cell lysates were centrifuged (4 °C, 12 000 r/min, 15 min) and incubated with 1 μL AMPK primary antibody (#5832, Cell Signaling Technology, Boston, USA) and 20 μL Protein A Magnetic Beads (MedChemExpress) overnight at 4 °C. The next day, immunocomplexes were washed with lysis buffer, centrifuged, and resuspended in an appropriate 1× loading buffer. Then, the protein samples were detected by western blot as described above.

### Dual-luciferase assay

HEK 293T cells were seeded into 12-well plates (1 × 10^5^) and transfected with the *Kidins220*- promoter-luciferase plasmid (Vigene Biosciences) and the renilla reniformis luciferase plasmid (Vigene Biosciences). Then, transfected NPCs were treated with different concentrations of CCG for 24 h. The Dual-Luciferase Reporter Assay System kit (Promega, WI, USA) was used to activate the activities of luciferase, which were detected by a microplate reader (BioTek, VT, USA).

### Immunofluorescence (IF) assay

NPCs were seeded into soft and rigid hydrogels and received different concentrations of CCG treatment for 24 h. Then, the cells were fixed with 4% polyformaldehyde (PFA, Servicebio, Wuhan, China), permeabilized with 0.2% Triton X-100 (BioFroxx), blocked with 1% BSA, and incubated with MRTF-A primary antibody (1:200, 21166-1-AP, Proteintech, Wuhan, China) overnight. The next day, the cells were immunoblotted with Cy3-secondary antibody (Boster) for 1 h and DAPI (Boster) for 10 min. The IF images were captured using an EVOS FL Auto Imaging System (Thermo Fisher Scientific). In addition, NPCs transfected with OE-Kidins220 for 24 h were subjected to IF assay as described above and incubated with p-AMPKα primary antibody (1:200, #50081, Cell Signaling Technology) overnight and immunoblotted with FITC-secondary antibody (Boster) the next day.

### Real-time PCR analysis

NPC RNA was extracted using Trizol reagent (Takara Bio Inc., Beijing, China). After purification, mRNA was reverse transcribed to cDNA using reverse transcriptase (Toyobo, Shanghai, China) and subsequently amplified using the primers (Supplementary Table 2) and SYBR Premix Ex Tap (Toyobo). The fluorescence signals were measured by an RT-PCR detection system (Bio-Rad), and the mRNA expression levels of the target genes were normalized to β-actin expression levels and analyzed using the 2^−ΔΔCt^ method.

### Chromatin immunoprecipitation (ChIP)

The ChIP Assay Kit (Beyotime, Shanghai, China) was used to obtain DNA-protein immune complexes. Briefly, chondrocytes were cross-linked with 1% formaldehyde, and chromatin fragmentation was performed by micrococcal nuclease. The chromatin solution was incubated with MRTF-A antibody (1 μg, 21166-1-AP, Proteintech) or negative control IgG antibody (30000-0-AP, Proteintech), then co-incubated with protein A/G magnetic beads overnight. The DNA-protein immune complexes were obtained according to the protocal, and the eluted DNA was next subjected to amplification. The primer set of GTGTCAGGAGGCCCTTTGAA (Forward) and GCCCTTCCAGACTTTGCTCT (Reverse) were used for further ChIP-PCR analysis of Kidins220 promoter regions.

### IVDD rat model generation and treatment

The effect of MRTF-A on IVDD progression was evaluated in two separate in vivo experiments. On the one hand, AAV-MRTF-A was used to up-regulate MRTF-A expression levels in NP tissues. Twelve rat tail IVD were subjected to the same treatment: Co6/7 disc was exposed and followed by suture as SHAM treatment; Co7/8 and Co8/9 disc were injected with AAV-NC and AAV-MRTF-A by a 31-gauge Hamilton syringe (Hamilton, NV, USA), respectively. On the other hand, CCG was used to inhibit MRTF-A in degenerative NP tissues. Thirty-six rats were randomly divided into three groups: the SHAM group (*n* = 12) received sham surgery as the control, whereas the IVDD group (*n* = 12) NP tissue was punctured by an 18-gauge outer diameter needle for 10 s and rotated twice; the CCG group NP tissue received 2 μL of CCG (2.5 µg/µL) injection after disc puncture and was subsequently subjected to CCG (2.5 mg/mL, 7.5 mg/kg) intraperitoneal injection weekly. At 4 and 8 weeks after surgery, imaging analysis and histological staining were performed on the rat’s IVD tissues in both experiments.

### Magnetic resonance imaging (MRI)

The rats were anesthetized with a 2% isoflurane/oxygen mixture and placed on the scan table in a prone position with the tail straight. MRI scanning was performed using a UNITED IMAGING 3.0-TMR scanner (Siemens, Shanghai, China). Twenty consecutive sagittal and coronal T2-weighted images were obtained by scanning the IVD tissues of the rat with a double-tuned-volume radiofrequency coil. MRI images were analyzed using the modified Pfirrmann score.^68^

### Micro-CT and X-ray

The rats were euthanized, and tail IVDs were collected for Micro-CT scan. Micro-CT scanning was performed using a micro-CT 50 system (Scanco Medical, Zurich, Switzerland), and the scanning parameters were set as: voxel size of 10 µm resolution, voltage of 100 kV, and a current of 98 µA. The CT three-dimensional and X-ray images were obtained using the evaluation system built into the microcomputed tomography system. The X-ray image was analyzed by disc height index (DHI) as previously described.^69^

### Histological staining and immunohistochemistry (IHC) assay

The rat’s tail IVD tissues were collected, fixed with 4% PFA, dehydrated, embedded in paraffin wax, and cut into 4 μm sections for histological staining. The structure and morphology of IVDs were observed by Hematoxylin-Eosin (HE, Servicebio) and Safranin O/Fast Green (Servicebio) staining according to the manufacturer’s instructions. After blocking and primary antibody incubation (Table S1), the sections were immunoblotted with secondary antibodies (Boster) and stained with DAB solution (Servicebio). Bright-field images were captured using an EVOS FL Auto Imaging System. In addition, IVD morphology was analyzed and evaluated by histological staining score.^70^

### GC-MS analysis

Treated NPCs were quick-frozen, grinded, and dissolved in 70% methanol. Gas chromatography-mass spectrometry (GC-MS) was used to identify 29 kinds of energy metabolic substances and products in cell samples (Table S3), and the charge-mass ratios of different substances were obtained. Finally, the peaks were corrected by Labsolution GC-MS software, and the area of peaks and relative content of substances were obtained by intelligent integration and manual correction.

### RNA sequencing

Total RNA was extracted by Trizol, evaluated by the Agilent 2100 Bioanalyzer (CA, USA), and purified for library preparation and sequencing on an Illumina Hiseq platform. Differential expression analysis was performed using the DESeq2 R package (1.20.0), and genes were considered significantly differentially expressed when the *P*-value was less than 0.05. Gene Ontology (GO) and Kyoto Encyclopedia of Genes and Genomes (KEGG) pathway analyses were performed using the clusterProfiler R package.

### Detection of glycolysis indicators

ATP level was evaluated by the ATP Colorimetric Assay Kit (Biovision, SF, USA). NPCs (5 × 10^5^) were extracted, the lysate was centrifuged, and the supernatant was collected for subsequent reactions according to the manufacturer’s instructions. The absorbance was evaluated at 570 nm (BioTek). In addition, pyruvate level was evaluated by the Pyruvate Colorimetric Assay Kit (Biovision), and the procedure was similar to ATP level detection.

Lactic acid level was evaluated using the Lactate Assay Kit II (Biovision). Treated NPC culture medium was replaced by serum-free medium, and supernatants were collected after 1 h. Then, the supernatants were mixed with reagents for 30 min and the absorbance was evaluated at 450 nm (BioTek).

Glucose concentration was determined by the Glucose Assay Reagent Kit (Beyotime). The supernatant deriver from treated NPCs was collected for subsequent reactions compared to fresh medium. The absorbance was evaluated at 630 nm (BioTek).

### Statistics

The statistical analyses and graphs were performed and generated using GraphPad Prism 8 software. The experimental data between multiple groups were compared using one-way analysis of variance and presented as the mean ± standard deviation. The ordinal variables, including histological score and Pfirrmann score, were analyzed by the Kruskal–Wallis H test. Statistical significance was set at *P* < 0.05.

## Supplementary information

Supplementary Material

Editorial Policy Checklist

## Data Availability

The datasets generated and/or analyzed during the current study are not publicly available due to be used for follow-up studies but are available from the corresponding author on reasonable request. Correspondence and requests for materials should be addressed to J.D. and L.S.
