# Supplementary material for: Matrix stiffness regulates nucleus pulposus cell glycolysis by MRTF-A-dependent mechanotransduction
Source: Bone Res. 2025 Feb 14;13:23. doi: 10.1038/s41413-025-00402-7 (PMC11828926; doi:10.1038/s41413-025-00402-7)
Supplement: Supplementary file 1 — Supplementary Material [file 41413_2025_402_MOESM1_ESM.doc]

**Supplementary Material**

**
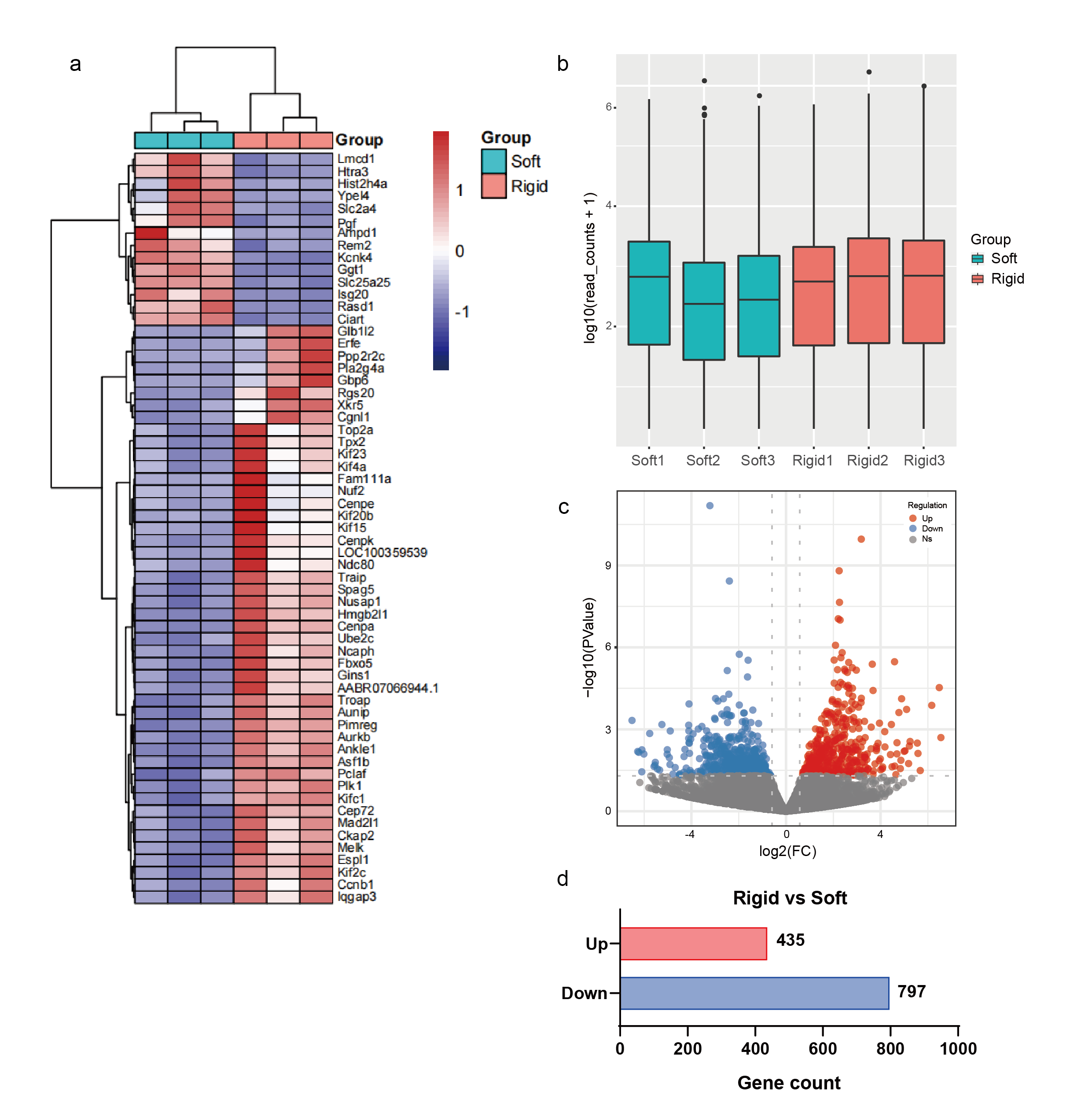
**

**Fig. S1: RNA sequencing results of NPCs under soft and rigid substrate incubation. (a)** Heat map of significantly differentially expressed genes (TOP 60) in NPCs cultured on soft or rigid substrates (Foldchange > 1.5, *P*-value < 0.05). **(b)** Comparison of overall gene expression (read count) for each sample. **(c, d)** The volcano plot shows significantly different genes (Rigid vs. Soft), with a total of 435 up-regulated and 797 down-regulated genes.

**
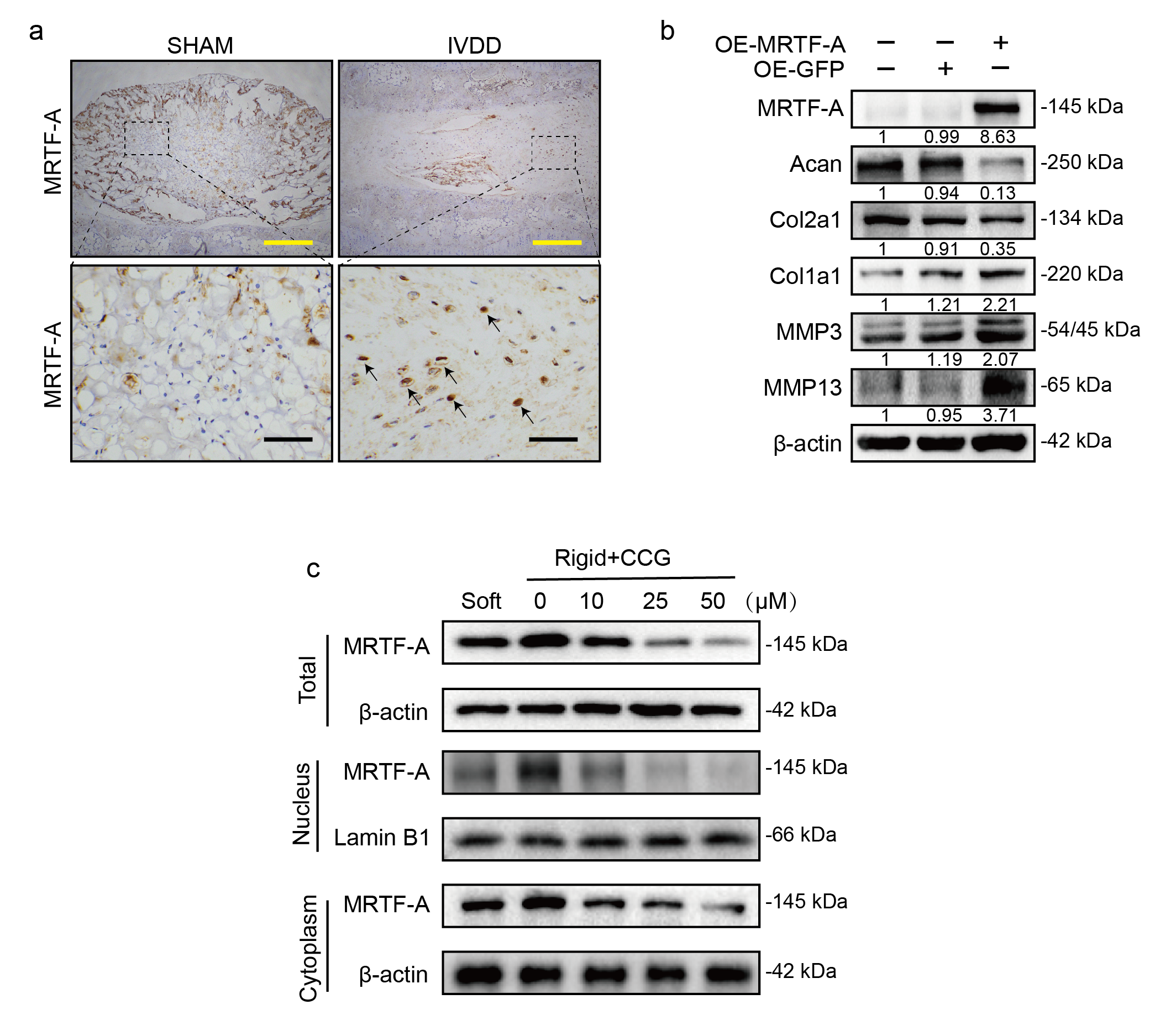
**

**Fig. S2: (a)** IHC staining of MRTF-A in NP tissues from SHAM and IVDD rat models (black arrows indicate representative positive cells). **(b)** The levels of MRTF-A, Acan, Col2a1, Col1a1, MMP3, and MMP13 in NPCs treated with OE-GFP or OE-MRTF-A for 24 h. **(c)** The level of MRTF-A in the nucleus and cytoplasm of NPCs treated with 0, 10, 25, and 50 μM CCG and cultured on soft or rigid substrates for 24 h. Yellow scale bar = 1000 μm and black scale bar = 200 μm.


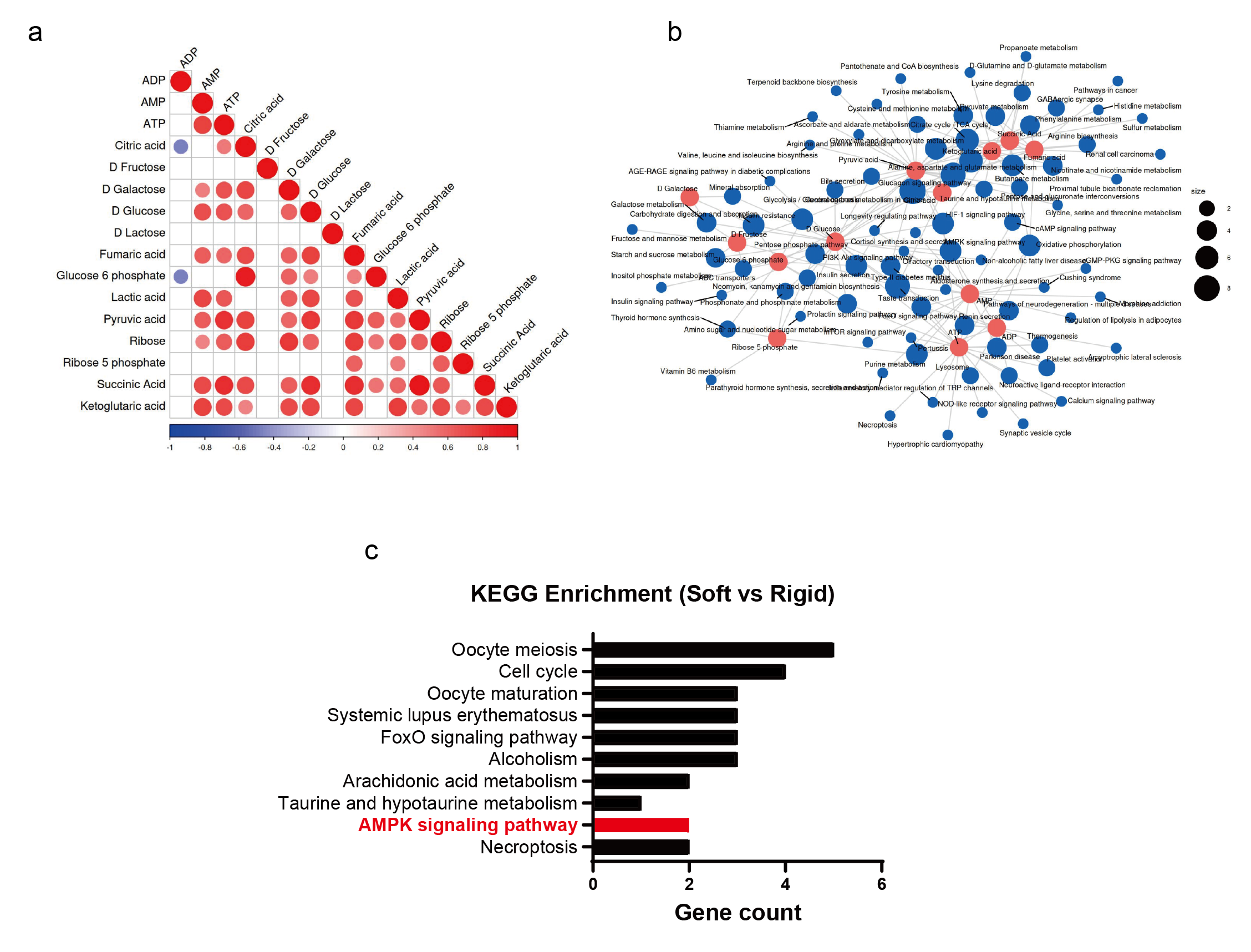


**Fig. S3: (a)** Differential metabolite association analysis map of GC-MS analysis results. **(b)** Association network diagram of the KEGG pathway and metabolites enriched in corresponding pathways. **(c)** KEGG enrichment analysis in NPCs cultured on soft or rigid substrates (top 10).

**
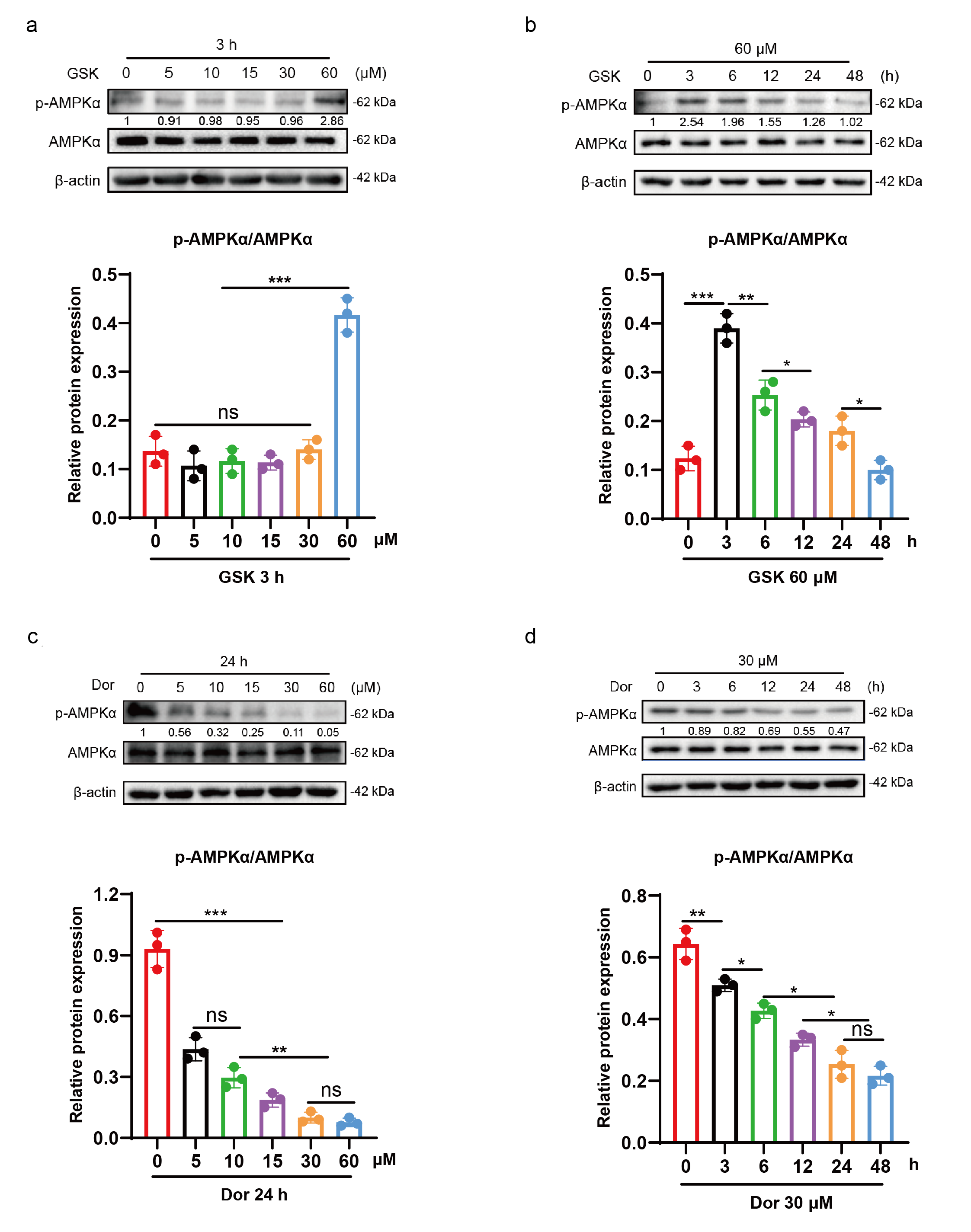
**

**Fig. S4: Concentration and duration of treatment of AMPK agonists (GSK) and inhibitors (Dor) in NPCs. (a)** The levels of p-AMPK and AMPK in NPCs treated with different concentrations of GSK for 3 h. **(b)** The levels of p-AMPK and AMPK in NPCs treated with 60 μM GSK for different durations (0-48 h). **(c)** The levels of p-AMPK and AMPK in NPCs treated with different concentrations of Dor for 24 h. **(d)** The levels of p-AMPK and AMPK in NPCs treated with 30 μM Dor for different durations (0-48 h). **P* < 0.05, ***P* < 0.01, ****P* < 0.001.

**
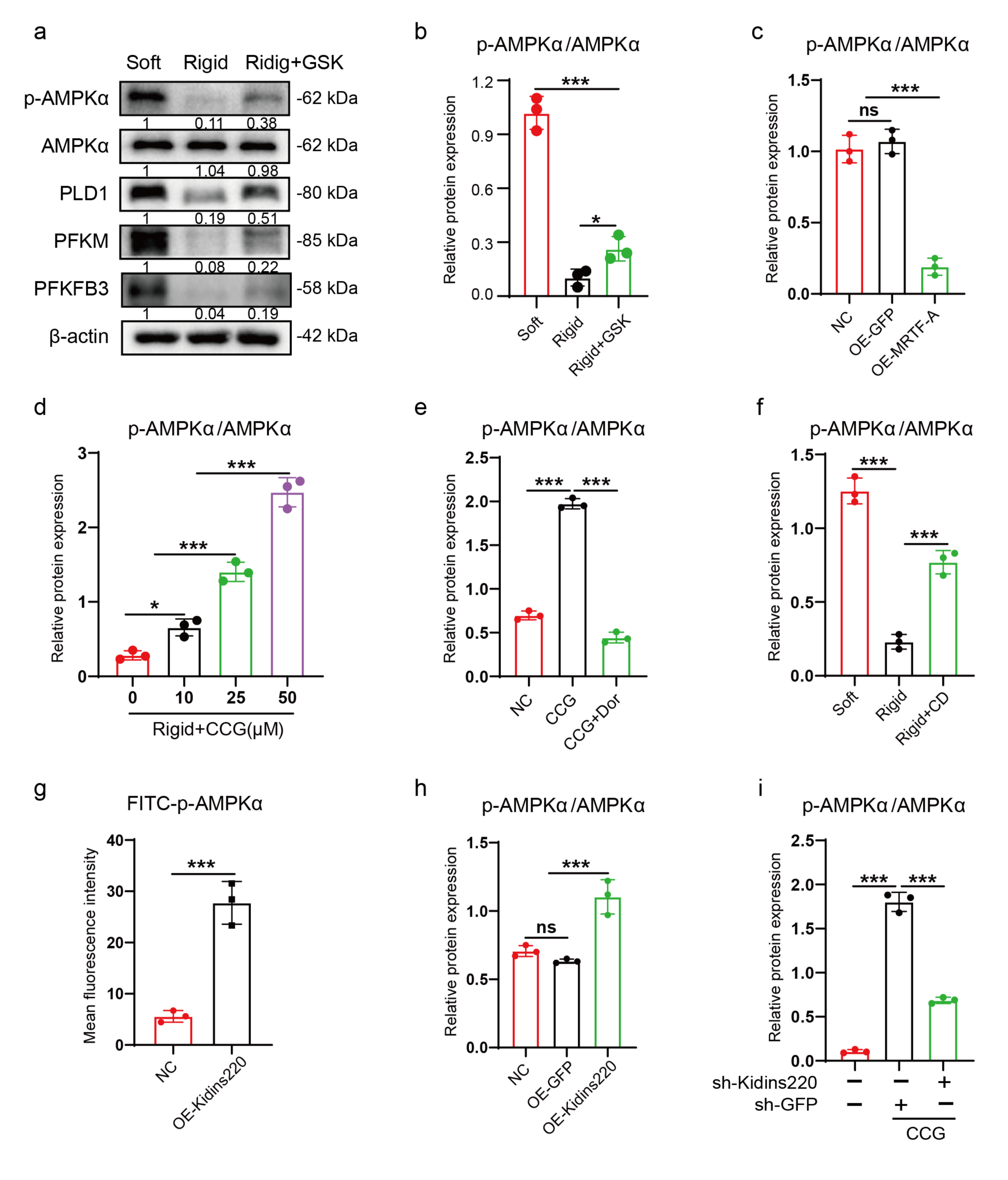
**

**Fig. S5:** **(a)** The levels of p-AMPKα, AMPKα, PLD1, PFKM, and PFKFB3 in NPCs treated with 60 μM GSK for 3 h under rigid substrate condition. The quantification of p-AMPKα/ AMPKα under different treatment: **(b)** rigid substrate and GSK, **(c)** OE-GFP or OE-MRTF-A, **(d)** different concentrations of CCG, **(e)** CCG and Dor, and **(f)** rigid substrate and CD treatment. **(g)** The evaluation of mean fluorescence intensity of p-AMPK treated with OE-NC or OE-Kidins220. The quantification of p-AMPKα/ AMPKα in NPCs treated with **(h)** OE-Kidins220 or **(i)** CCG combined with sh-Kidins220. **P* < 0.05, ****P* < 0.001.

**
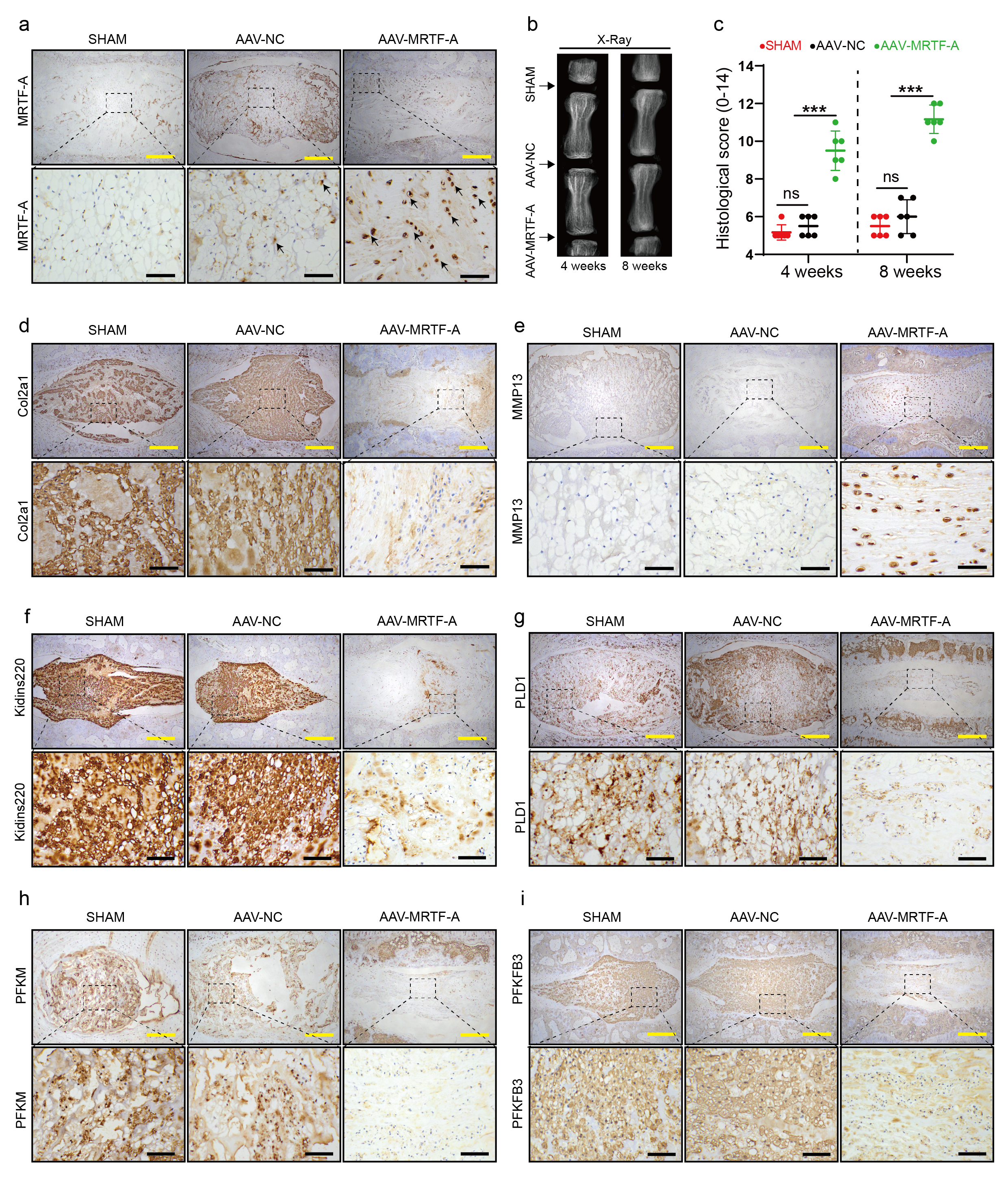
**

**Fig. S6: (a)** IHC staining of MRTF-A in NP tissues after AAV-NC and AAV-MRTF-A injection (black arrows indicate representative positive cells). **(b)** The X-ray images of rat intervertebral discs after AAV-NC and AAV-MRTF-A injection. **(c)** The histological staining score quantification of IVD image. IHC staining of **(d)** Col2a1, **(e)** MMP13, **(f)** Kidins220, **(g)** PLD1, **(h)** PFKM, and **(i)** PFKFB3 in NP tissues after AAV-NC and AAV-MRTF-A injection (yellow scale bar = 1000 μm and black scale bar = 200 μm).

**
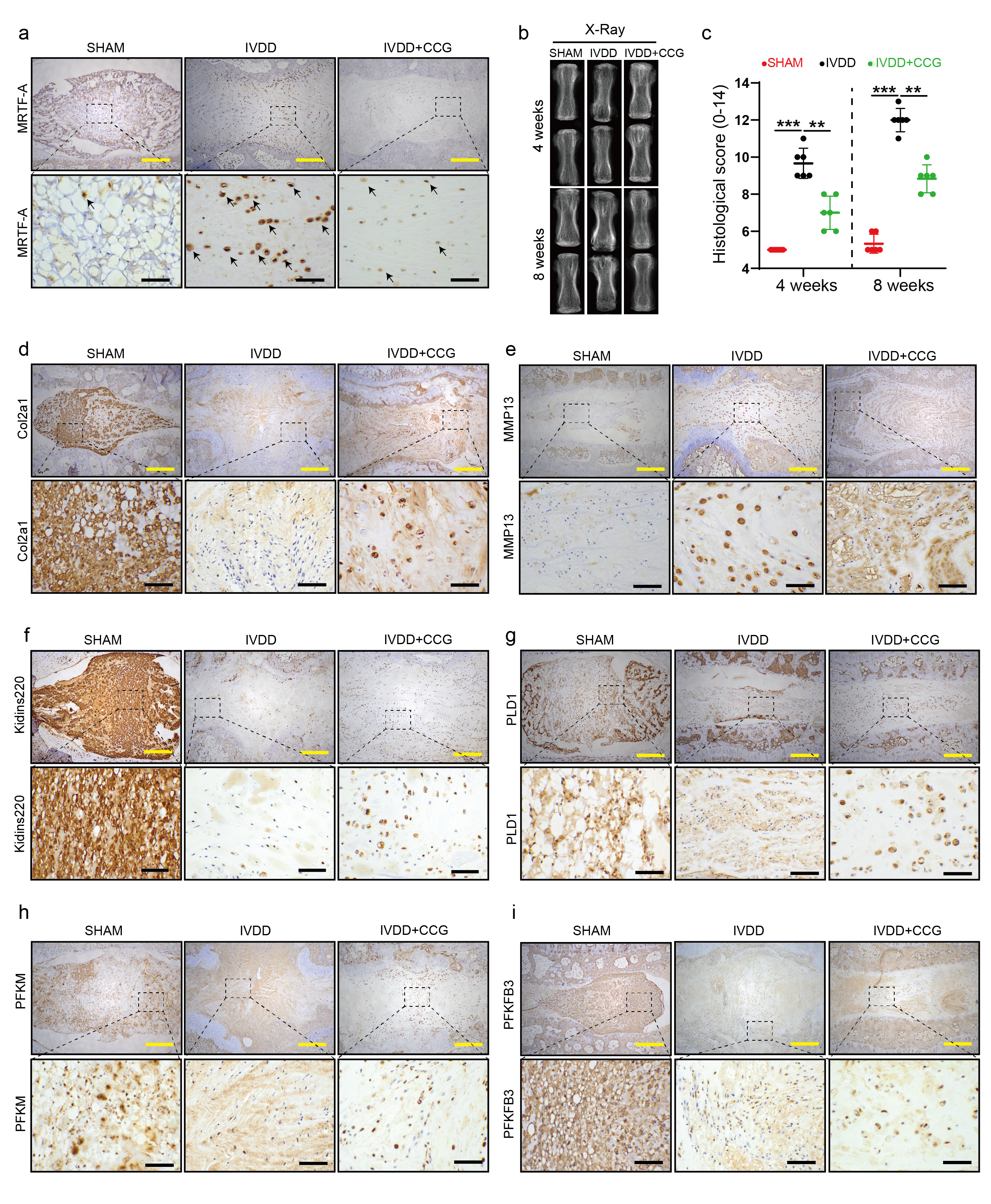
**

**Fig. S7: (a)** IHC staining of MRTF-A in NP tissues after IVDD surgery and CCG injection (black arrows indicate representative positive cells). **(b)** The X-ray images of rat intervertebral discs after disc puncture combined with CCG treatment. **(c)** The histological staining score quantification of IVD image. IHC staining of **(d)** Col2a1, **(e)** MMP13, **(f)** Kidins220, **(g)** PLD1, **(h)** PFKM, and **(i)** PFKFB3 in NP tissues after IVDD surgery and CCG injection (yellow scale bar = 1000 μm and black scale bar = 200 μm).

| **Supplementary** **Table 1.** Primary antibodies used in the western blot and IHC experiments | | | | |  |
| --- | --- | --- | --- | --- | --- |
| **Name** | **Catalog Number** | **Dilution Ratio** | **Application** | **Source** | |
| β-actin | 66009-1-Ig | 1:10,000 | WB | Proteintech | |
| Acan | 13880-1-AP | 1:1,000 | WB | Proteintech | |
| Col2a1 | 28459-1-AP | 1:1,000 | WB | Proteintech | |
| Col1a1 | #84336 | 1:1,000 | WB | Cell Signaling Technology | |
| MMP3 | 17873-1-AP | 1:500 | WB | Proteintech | |
| MMP13 | 18165-1-AP | 1:1,000 | WB | Proteintech | |
| PLD1 | 12148-1-AP | 1:1,000 | WB | Proteintech | |
| PFKM | 55028-1-AP | 1:1,000 | WB | Proteintech | |
| PFKFB3 | 13763-1-AP | 1:1,000 | WB | Proteintech | |
| MRTF-A | 21166-1-AP | 1:1,000 | WB | Proteintech | |
| Lamin B1 | 12987-1-AP | 1:1,000 | WB | Proteintech | |
| p-AMPKα | #50081 | 1:1,000 | WB | Cell Signaling Technology | |
| AMPKα | #5832 | 1:1,000 | WB | Cell Signaling Technology | |
| Kidins220 | 21856-1-AP | 1:1,000 | WB | Proteintech | |
| MRTF-A | 21166-1-AP | 1:200 | IHC | Proteintech | |
| PLD1 | 12148-1-AP | 1:200 | IHC | Proteintech | |
| PFKM | 55028-1-AP | 1:200 | IHC | Proteintech | |
| PFKFB3 | 13763-1-AP | 1:200 | IHC | Proteintech | |
| Col2a1 | 28459-1-AP | 1:200 | IHC | Proteintech | |
| MMP13 | 18165-1-AP | 1:200 | IHC | Proteintech | |
| Kidins220 | 21856-1-AP | 1:200 | IHC | Proteintech | |

| **Supplementary** **Table 2.** Primer sequence used in the PCR experiment | |
| --- | --- |
| **Gene** | **RNA sequence** |
| Rat-*Kidins220*-F | 5’-ATGAGAGGAACGAGTGTGGC-3’ |
| Rat-*Kidins220*-R | 5’-TGGATGTGCCCCTCCTTAGA-3’ |
| Rat-*β-actin*-F | 5’-CCCGCGAGTACAACCTTCTTG-3’ |
| Rat-*β-actin*-R | 5’-GTCATCCATGGCGAACTGGTG-3’ |
| F: Forward; R: Reverse | |

| **Supplementary** **Table 3.** Metabolites analyzed in GS-MC | | | |
| --- | --- | --- | --- |
| **Name** |  |  |  |
| Galactose | L-lactic acid | Ribose-5-phosphate | Malic Acid |
| α-Ketoglutaric acid | Sodium pyruvate | Fructose-6-phosphate | Succinate |
| 2-Deoxy-D-ribose | 3-phosphoglycerate | Fructose 1,6-bisphosphate | Fumarate |
| Glucose | Diacetone phosphate | Erythrose 4-Phosphate | Citric acid |
| Lactose | Phosphoenolpyruvate | Adenosine triphosphate | Ribose |
| Lactic acid | Glucose-6-phosphate | Adenosine diphosphate | NADPH |
| Fructose | D-Glucose-6-phosphate | Adenosine monophosphate | NADP+ |
| Glutathione |  |  |  |
